# Developing a realistic sexual network model of chlamydia transmission in Britain

**DOI:** 10.1186/1742-4682-3-3

**Published:** 2006-01-20

**Authors:** Katherine ME Turner, Elisabeth J Adams, Nigel Gay, Azra C Ghani, Catherine Mercer, W John Edmunds

**Affiliations:** 1 Health Protection Agency, Centre for Infections, 61 Colindale Ave, Colindale, London, NW9 5EQ, UK; 2 London School of Hygiene & Tropical Medicine, Keppel Street, London WC1E 7HT, UK; 3 Department of Primary Care and Population Sciences, University College London, Mortimer Market Centre, Mortimer Market, London WC1E 6AU, UK

## Abstract

**Background:**

A national chlamydia screening programme is currently being rolled out in the UK and other countries. However, much of the epidemiology remains poorly understood. In this paper we present a stochastic, individual based, dynamic sexual network model of chlamydia transmission and its parameterisation. Mathematical models provide a theoretical framework for understanding the key epidemiological features of chlamydia: sexual behaviour, health care seeking and transmission dynamics.

**Results:**

The model parameters were estimated either directly or by systematic fitting to a variety of appropriate data sources. The fitted model was representative of sexual behaviour, chlamydia epidemiology and health care use in England. We were able to recapture the observed age distribution of chlamydia prevalence.

**Conclusion:**

Estimating parameters for models of sexual behaviour and transmission of chlamydia is complex. Most of the parameter values are highly correlated, highly variable and there is little empirical evidence to inform estimates. We used a novel approach to estimate the rate of active treatment seeking, by combining data sources, which improved the credibility of the model results. The model structure is flexible and is broadly applicable to other developed world settings and provides a practical tool for public health decision makers.

## Background

Chlamydia is a very common, curable sexually transmitted infection (STI) caused by the *Chlamydia trachomatis *bacteria. Chlamydia prevalence in young women attending general practice in Britain was estimated to be 8.1% in those under 20 and 5.2% in those aged 20–24 [1], and is similar in other developed countries. Many infections are asymptomatic, resulting in a large reservoir of undetected, untreated infections [2]. Untreated chlamydia infection may result in long-term sequelae in women including pelvic inflammatory disease (PID) and ectopic pregnancy [3]. Detection of chlamydia has become easier with the recent introduction of rapid, sensitive, affordable, and non-invasive DNA tests [4]. Treatment is also straightforward and inexpensive with doxycycline or azithromycin [5]. Chlamydia screening therefore, has been or is being implemented in various developed countries including USA, Sweden, Netherlands, and UK [6-9]. However much of the epidemiology of chlamydia remains poorly understood [10] and there are many questions regarding the long term impact of interventions, such as how much PID is attributable to chlamydia infection and what are the economic and health costs and benefits of chlamydia screening? Appropriate mathematical models are required to address these questions adequately. Models are able to compare a variety of "what if" scenarios and inform estimates of biological and epidemiological parameters which are difficult to measure in practice e.g. transmission rate or the proportion of symptomatic cases seeking treatment.

Population-based deterministic models were first used to illustrate the importance of the contact structure and dynamic aspects of infection [11-13]. However population-based models fail to capture important individual level effects in the sexual network. For example, re-infection is dependent on the infection and treatment status of current partners, not the average level of infection in the community. Individual based models of STI transmission with dynamic sexual partnerships have been developed which can incorporate such effects [14,15]. Ghani *et al *developed an individual-based, dynamic sexual network model of gonorrhoea transmission within a highly active "core-group" population [15]. Individuals and their partnerships are explicitly represented, enabling detailed analysis of the network structure. Partnerships form according to mixing preferences based on sexual activity level and dissolve dynamically.

There is a growing public health need for a realistic, dynamic model of chlamydia transmission to inform and interpret the potential effect of interventions such as screening programmes and partner notification [16] To this end it was necessary to extend Ghani's model. The distribution of chlamydia is more widespread and less focussed in core groups than gonorrhoea, so a population model was developed [17]. The US Add Health study found a ten-fold higher prevalence of chlamydia (4.19%) compared with gonorrhoea (0.43%) in a probability sample of 18–26 year olds [2]. In the UK there were 104,155 chlamydia diagnoses in GUM clinics in 2004, compared with 22,335 of gonorrhoea [18] To be realistic, the model also requires age-structure, because chlamydia prevalence declines with increasing age [1], and at the population level sexual behaviour and partner choice are strongly age-dependent [19,20]. Therefore, we extended the model to incorporate age-structured sexual behaviour and partnership preferences in the general population. The final model is a realistic representation of sexual behaviour and chlamydia epidemiology in England, but is also broadly applicable in other developed world settings.

The purpose of this paper is to describe the model parameterisation method and to present the values of selected parameters that will be used in future applications to explore chlamydia screening interventions.

## Method

### Model description

The model is a stochastic, individual based network model based on that described by Ghani *et al *[15]. It is exclusively heterosexual and includes dynamic partnership choice, formation and dissolution, disease transmission, and recovery. The model has a **S**usceptible-**I**nfected-**S**usceptible (SIS) structure. Susceptible individuals are infected, then either seek care or remain untreated, returning to a susceptible state following spontaneous recovery or treatment. The extended model also incorporates age-structured sexual behaviour and mixing, screening, and partner treatment. The resulting complex model can simulate a range of sexual behaviour, disease transmission and control programmes. The model simulates sexual behaviour, chlamydia transmission and interventions in Britain.

The parameterisation of sexual behaviour was primarily informed by the National Survey of Sexual Behaviour and Lifestyles (Natsal) 2000 [19,21,22], a stratified, nationally representative, probability sample survey of men and women in Britain aged 16–44. Over 12,000 individuals in the core sample, including an ethnic minority boost sample, were asked about their sexual behaviour via face-to-face interview and computer assisted self-interview ('CASI') [23]. The response rate was 65.4% in the core sample and 63.0% in the ethnic minority boost sample.

### Sexual behaviour

Individuals are explicitly represented in the model by age, gender, preferred number of partners, preferred duration of partnerships, identity of current and past partners, infection status (and whether actively seeking treatment or not), and other clinical characteristics such as number of screens and results. For ease of analysis, behavioural data equivalent to Natsal 2000 [19,21,22]questionnaire responses (including partners in the last year and new partners in the last year) were also stored for each individual.

The rate of sexual partner change for an individual is determined by the rate of new partnership formation, the availability of suitable partners, the rate at which partnerships dissolve, and the gap between partnerships. Individuals are available to form a new partnership if their current number of partnerships is less than their desired number of partnerships (either 1 or 2). Potential pairs are selected at random from the pool of available candidates and the partnership forms stochastically according to probabilities assigned in age mixing matrices for men and women (derived from Natsal 2000 data). Most partnerships form between people of the same age and men have a tendency to form partnerships with women somewhat younger than themselves (age difference mode = 0 years, mean = 2) [19]. The duration of partnerships is assumed to be exponentially distributed, giving a constant per time-step probability of a partnership dissolving of 1/(average duration of partnership). Long and short partnerships have different mean durations (Table 1). When a new partnership forms in the model, one person from the pair is selected at random and that person's preferred duration (long or short) is assigned to the new partnership. This means that those who prefer long partnerships sometimes have short partnerships, and vice versa. There is a gap between partnerships, during which time an individual cannot form any new partnerships, plus an additional period of time when an individual cannot form a partnership with their most recent partner to prevent the same partnership reforming immediately the pair become available.

**Table 1:** Fixed model parameters

**Parameter**	**Best fit or estimated value**	**Source**
**Behavioural parameters**		
Population size (Female = 20,000, Male = 20,000)	40,000	-
Age range in years (uniform distribution)	16–44	Natsal 2000 [19]
Preferred number of concurrent partners		Natsal 2000 [19]
<35 years old	1 or 2	
35+ years old	1	
Proportion wanting 2 partners (< 35 years old)	0.05	Assumption based on Kretzschmar model [24]
Mean duration of short partnerships (days)	14	Assumption based on Natsal 2000 [19]
Number of sex acts per day		Assumption based on Kretzschmar model [24]
Short partnerships	1	
Long partnerships	0.25	
Mean gap in days between partnerships (dispersion)*	14 (2)	Assumption
**Infection parameters**		
Duration (in days)		Assumption based on Golden [10], Korenromp [30]
No treatment seeking	180	
Treatment seeking	30	
Mean refractory period (in days) following treatment (dispersion)*	7 (10)	Assumption based on CEG guidelines [5]
**Health care parameters**		
Attendance rate at health care setting (proportion who report attending a health care setting in the last 12 months)	0.85	Chlamydia Recall Study [26,27]
Treatment efficacy (in those partner notified or screened)	0.95	Treatment guidelines [37]
Mean delay (in days) before partner treatment (dispersion)*	7 (10)	Assumption based on unpublished Recall study
Probability of accepting screen	0.5	Assumption based on screening studies [38,39]

*Parameters drawn from a negative binomial distribution, mean and dispersion.

The level of concurrency is defined as the proportion of the population that prefer 2 partners until they reach 35 years of age, fixed at 5% in these simulations (Table 1). After age 35, all persons prefer one sex partner [24], although existing partnerships are not ended. If either partner has an existing partner when the partnership forms, the concurrent partnership is always assigned as short.

### Age dependent processes

Age is an important determinant of sexual behaviour and chlamydia risk [19-21]. The model population is aged 16–44, as in Natsal 2000. Aging occurs deterministically once per year for all individuals in the population. The preferences for new partnerships (but not existing partnerships) are adjusted annually. When an individual reaches age 45, they are removed from the population and a new 16 year old enters (gender maintained). Existing partnerships are not ended, but are flagged as external to the population, so that individuals <45 year of age in a stable partnership do not become prematurely available for new partnerships when their partner passes 45 years of age.

In the model, sexual partnerships form stochastically according to age mixing preferences. Individuals generally form fewer new partnerships as they age. This is implemented by a fraction of the population who prefer short partnerships switching to long, all those who prefer long partnerships increasing the average duration of partnerships (i.e. decreasing the chance of the partnership dissolving) and shifting the preference for partners of different ages according to the age mixing matrices.

### Infection processes

Transmission of chlamydia occurs stochastically between an infected index case and uninfected current partner, with a per sex act probability, assuming one sex act per day in partnerships which have lasted less than one month and 0.25 per day in longer partnerships.

There is a constant per day probability of recovery of (1/average duration of infection). A fraction of newly infected individuals are assumed to actively seek treatment and to recover at a faster rate than those not seeking treatment. The recovery rate of those not seeking care is influenced by the level of screening and partner notification. After treatment for any reason, individuals enter a variable refractory period during which re-infection cannot occur, to simulate patients following advice to abstain for a week and until partners have been treated (British Association of Sexual Health and HIV (BASHH) guidelines) [5].

### Partner notification and screening

Partner notification is implemented by examining partnerships within the last 3 months (as per BASHH guidelines) [5]. For each partner there is a probability of being contacted. Notified partners are treated after a variable delay following treatment of the index case, with certain efficacy. Individuals may be partner notified as a result of the index seeking treatment due to symptoms or screening. For individuals treated via partner notification, their partners are not traced.

Various screening programmes can be implemented in the model, some of which are explored in Turner *et al *(Turner KME, Adams EJ, LaMontagne DS, Emmett L, Baster K, Edmunds WJ. **Modelling the effectiveness of chlamydia screening in England **(*submitted*). Available upon request).

### Model parameterisation

For many of the model parameters few data are available (e.g. fraction of individuals who seek treatment for infections), the value is highly variable (e.g. duration of untreated infection [10,25]) or the parameter of interest cannot be measured directly (e.g. sexual behaviour is usually collected retrospectively and cross-sectionally as number of partners over a given time period, but is implemented prospectively as desired partner formation and dissolution rates). Therefore, some of the parameters are estimated by fitting the model to data.

Behavioural parameters were informed principally by Natsal 2000 [19,21,22].Infection and treatment parameters were fitted using Natsal 2000 and other available data sources [1,21,26,27].

### Behavioural parameter estimation

Estimation of behavioural parameters was done in two stages: an exploratory stage, to assess the impact of different parameters on model behaviour and to refine parameter ranges, followed by a second phase of fitting using maximum likelihood. Several parameters were unknown:

• the proportion of individuals desiring short partnerships (males (M) and females (F))

• the proportion of individuals changing from wanting short partnerships to long partnership each year (M, F)

• the average duration of long partnerships (M, F)

• the annual increase in preferred partnership duration (M, F)

• the duration of the average gap between partnerships.

Sexual behaviour stabilised after running the model for 10 years, and a population of 6000 (3000 males and females) was sufficient to generate the range of behaviour observed in larger model populations. Latin hypercube sampling (LHS) was used to generate more than 800 parameter sets in the exploratory phase. The average of 5 model realisations was used to maximise efficiency. There was high correlation between the parameters in determining the fit of the model.

The model outputs were grouped by age, sex and sexual activity and were compared to Natsal 2000 data. Sexual activity groups were defined on the basis of number of partners (0–1, 2–3, 4–7, 8+) and were populated with either the number of individuals reporting that activity level (i.e. frequency) or the number of partnerships contributed by individuals within that group (weighted frequency).

In the Natsal 2000 survey, there was inconsistency between genders in reported behaviour: men reported on average 1.5 times as many partners as women, in common with other such surveys [19,28]. During the exploratory phase, male and female data were therefore fitted separately, using least squares. Fitting to the male reported data generated higher rates of partner change than fitting to female data. Fitting to data on the number of partnerships generated higher rates of partner change than fitting to the number of individuals observed with different levels of activity.

For the second phase, the model was fitted using maximum likelihood to male partnerships in the last year only. This best replicated the variability and range of observed behaviour, giving a longer tail to the distribution (i.e. including a few individuals with many partners). It has also been suggested that male reporting may be more reliable than females [29].

**Figure 1:**
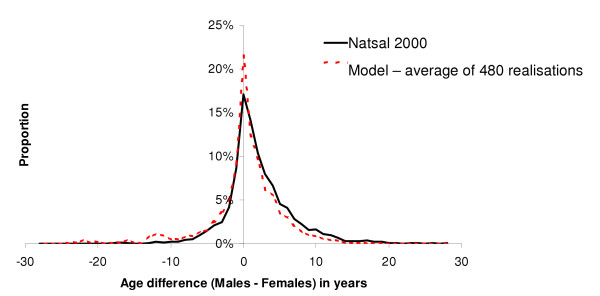
Frequency of age differences between sexual partners (males compared to females, aged 16–44) observed in Natsal 2000 and in the model.

Results from the exploratory runs showed that varying as few as four population parameters was sufficient to generate a range of sexual behaviour comparable with the empirical data. The proportion of short partnerships (M, F) at recruitment into the sexually active population and the proportion that change from preferring short to long partnerships (M, F) were therefore varied in the second phase. The remaining parameters were fixed (Table 1): average duration of long partnerships in 16 year olds, the annual increase in desired partnership duration, duration of short partnerships and the duration of the gap between partnerships. All fixed parameters were assumed to be the same for men and women. The log likelihood, saturated log likelihood and deviance were calculated (Appendix). Behavioural parameters and their best fit values are given in Table 2. A matrix of probabilities of partnership formation by age was derived from the age differences between sexual partners observed in Natsal 2000 data and used in the model. The age differences observed in the model are compared with Natsal 2000 in Figure 1.

**Table 2:** Fitted model parameters

**Parameter**	**Best fit or estimated value**	**Limits of 95% CI**	**Range (increment)**	**Source**
**Behavioural parameters**				
Proportion that switch from desiring short to long partnerships per year				Fitted to Natsal 2000 [19]
Men	0.04	0.02–0.06	0.0–0.08	
Women	0.08	0.06–0.08	0.04–0.12 (0.02)	
Initial proportion of 16 year olds desiring short partnerships				Fitted to Natsal 2000 [19]
Men	0.6	0.5–0.7	0.4–0.8	
Women	0.5	0.4–0.6	0.3–0.7 (0.1)	
Mean duration in days of long partnerships (16 year olds)	900			Based on exploratory fitting to Natsal 2000 [19]
Increase in partnership duration per year, in days	200			Based on exploratory fitting to Natsal 2000 [19]
**Infection parameters**				
Transmission probability per sex act	0.0375	0.035–0.04	0.035–0.05 (0.0025)	Fitted to Natsal 2000 [19] & Adams *et al *[1]
Proportion seeking treatment				Fitted to Natsal 2000 [19] & Adams *et al *[1]
Men	0.0	0.04–0.05	0–0.05	
Women	0.045	0–0.005	0–0.055 (0.005)	
**Health care parameters**				
Proportion of partners notified	0.2	0.1–0.25	0.0–0.5 (0.05)	Fitted to Natsal 2000 [21] & Adams *et al *[1]

Note: Fitted parameters are presented with the limits of the 95% confidence intervals (meaning that the 95% CI lies within those limits, further refinement was not done). The range tested in the fitting routines and the increment used is also shown.

### Infection parameter fitting

Chlamydia prevalence in the model depends on the transmission probability, duration of infection in those cases seeking treatment and not seeking treatment, the proportion seeking treatment, and the level of partner notification. Estimates for the duration of chlamydial infection vary greatly [10,30]. Further, the duration and transmission probability are highly correlated in determining chlamydia prevalence. We therefore chose to fix the average duration of infection in men and women at one month for those seeking treatment and six months for those not seeking treatment. The transmission probability, the proportion seeking treatment (M/F), and the level of partner notification were allowed to vary. Infection was introduced into the population and run for 15 years to reach a stable equilibrium, before calculating the model fit.

The model was fitted to data on chlamydia prevalence in women and the proportion of individuals who have reported ever having been diagnosed with chlamydia (and presumed treated), by age and gender [1,21]. Chlamydia prevalence estimates were taken from a systematic review of chlamydia prevalence in general practice (GP) clinic attendees [1]. These were estimated for various factors using a random effects regression model. Numerators and denominators were generated to ensure the prevalence and their 95% confidence intervals (CI) were the same as those in the systematic review [1]. Data on previous chlamydia diagnoses were obtained from the Natsal 2000 survey. Those older than 25 years reported less past treatment for chlamydia than younger women, which may reflect recent changes in testing, treatment, prevalence, or recall bias. Therefore data on previous diagnosis for males and females aged <25 years only and chlamydia prevalence in all age groups were used to fit the model. The binomial log likelihood, saturated log likelihood and deviance for each subgroup were calculated and then summed (Appendix).

Exploratory runs of the model were performed to predict the likely range of values for the varied parameters (each parameter set was averaged over 15 simulations). This range was then further refined by systematically combining parameters (proportion seeking treatment (M/F), transmission probability, and partner notification), by fixing two parameters and allowing the others to vary. Once a local best fit was found (lowest deviance), the other parameters were varied to search for a better fit. Thirty realisations were performed for each parameter set for the final fitting routines. Univariate sensitivity analysis was performed for each of the five parameters, and the 95% CI was estimated by finding those parameter values that lie within 3.84 of the deviance estimate.

## Results

**Figure 2:**
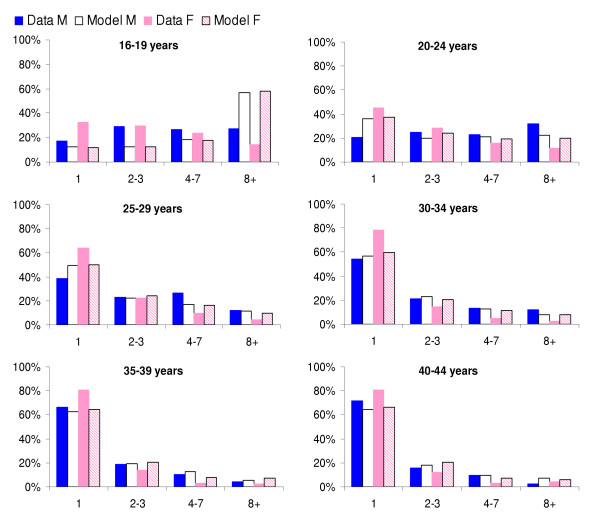
Proportion of partnerships contributed by different activity groups for the best fitting model (fitted to male partnerships), model output compared with Natsal 2000 data by age group and gender.

The results of fitting the model to behavioural data are shown in Figure 2 for male and female partnerships in the last year. The best fit parameter values, and the values that gave fits within 95% confidence limits are presented in Table 2. The model fits better to the male data than the female data, due to the choice of fitting procedure (i.e., the model was fitted to male behavioural data). In both males and females, the model overestimates the number of partners of the youngest age groups, and slightly underestimates in older age groups. The fitted model has a higher rate of partner change in females than observed in the data. The discrepancy between data and model is greatest in the youngest women

**Figure 3:**
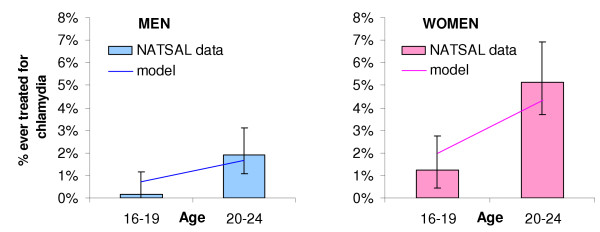
Baseline results for the proportion of males (a) and females (b) by age group ever treated for chlamydia, Natsal 2000 compared to the model.

**Figure 4:**
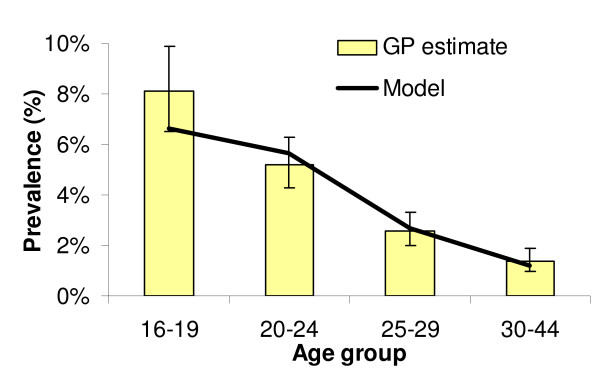
Baseline model chlamydia prevalence by age compared with estimated prevalence in general practice attendees (Adams *et al*, 2004) [1].

Given the set of behavioural parameters, the estimated biological parameters (and 95% confidence intervals) that produced the best fit are shown in Table 2. The best fitting model suggests a partner notification efficacy of 20%, per sex act transmission probability of 0.0375 and that a small fraction of cases are treated as a result of active treatment seeking (less than 5% of new female and 0.05% of new male cases). The best fitting model results are shown in comparison with the proportion reporting chlamydia treatment (Figure 3) and the prevalence of chlamydia in women (Figure 4).

## Discussion

The aim of this study was to develop a flexible, credible model of chlamydia transmission in Britain to address public health questions regarding chlamydia epidemiology and interventions including screening. We extended the model of Ghani *et al *to incorporate relevant features such as age-dependent sexual behaviour [15]. We used multiple data sources and an iterative process of parameter fitting and refinement to estimate sexual behaviour and biological parameters representative of current chlamydia epidemiology in Britain.

The distribution of sexual behaviour in the fitted model is broadly similar to that observed in Britain (Figure 2). In the model the total number of partnerships contributed by men and women are equal, because it is a closed population and partnerships can be counted perfectly. However, the model was fitted to male partnership data from Natsal 2000, which found that men report more partnerships than women [19,28]. Data available to validate and parameterise the model are based on retrospective accounts of individual's sexual behaviour, which are subject to various biases [31,32]. The reasons for the observed discrepancy are not fully understood, but could include male over-reporting, female under-reporting or gender differences in the distribution of partners. An Australian study compared reports of sexual behaviour under different survey conditions and found that males' reports were more consistent than females', and that females tended to report fewer partners when they believed the responses were not anonymous compared with when they believed lies would be detected, suggesting a bias towards underreporting [29]. Others have suggested that the difference between men and women primarily lies in the tail of the distribution and that female sex workers, who are likely to be poorly represented in population-based surveys, may supply the extra partnerships reported by men [33,34]. The true situation is probably a combination of these. We chose to fit the model to behaviour reported by men, as this may be more reliable. However, the sexual activity of women in the model is then higher than that reported in the data. The difference is greatest in the youngest women. If we had fitted to either women or some average of both, the model would have fitted neither data set well, although the overall model behaviour would be roughly similar and the fitted infection parameters would be slightly different.

The distribution of chlamydia by age and the number of people treated for infection follows that observed in young women [1,21]. Chlamydia prevalence is highest in the youngest age groups and lowest in the oldest. While surveillance data from genitourinary medicine clinics suggest that male prevalence may be highest in the 20–24 year old ages [18], a recent review does not suggest a difference in male and female prevalence, therefore we fitted to female data only. More data on the prevalence and incidence of chlamydia in men are needed to improve the parameter estimates [1].

The estimates of transmission probability are highly dependent on the values of the duration of infection chosen, but there are few reliable data on the timing of treatment or recovery under different scenarios of symptoms, contact tracing and screening. If the average duration of all infections were shorter than we modelled, the transmission probability would need to be higher to fit to the same overall prevalence. The level of partner notification (that is partners of contacts are known to have been tested and treated) predicted by the best fitting model was 20%. Data from the Chlamydia Recall Study suggested that partner notification might be as high as 50% in a study setting [26]. There are problems in interpreting the estimate of 20% as it is also correlated with the other infection parameter estimates and was fitted to the observed low rate of treatment. However the efficacy in a non-study setting is likely to be lower and the importance of maintaining and improving partner notification is crucial to the long-term success and effectiveness of interventions.

The proportion seeking treatment is low compared with other estimates of the proportion symptomatic [3,24,35]. This is due to several reasons. Firstly, active treatment seeking is not directly analogous to symptomaticity, which is an assumption in our model. A modelling study has suggested that the proportion of time an infection shows symptoms may be less frequent and also intermittent [30], and therefore may not prompt an individual to seek treatment if his/her symptoms disappear. In a recent US Add Health study, 4.19% of 18–26 year olds were infected with chlamydia, and more than 95% of infections were asymptomatic [2]. In the model, those who have reported treatment for chlamydia may have done so from either seeking treatment or through partner notification. In reality, treatment may be more frequent (with or without confirmed diagnosis) due to co-treatment of gonorrhoea cases or syndromic management of urethritis in men [36]. Secondly, we fitted to very low rates of treatment observed in the population, particularly among men, based on retrospective data collected by Natsal 2000. Recent data from the Health Protection Agency show that chlamydia diagnoses (and presumably treatment) have increased since 2000, from both a real increase in chlamydia prevalence and increased testing and diagnoses through education and screening [18]. We compared our estimates of treatment seeking to those in the model by Kretzschmar *et al *[24], which is the most thorough study published to date and is broadly comparable to ours in terms of structure and dynamics. We ran our model using the infection parameters from their published model, including a higher proportion of symptomatic infection (higher treatment rate). The model chlamydia prevalence was similar to that observed using our values, but the proportion of 20–24 year olds ever treated was over 45%. This compares with 4.5% in the fitted model and 5.1% (3.7–6.9%, 95% CI) of 20–24 year old women ever treated for chlamydia reported in Natsal 2000. Similarly, the Chlamydia Recall Study found that 8% of women aged 20–24 reported past treatment for chlamydia [26]. We believe that, although the true rate of treatment seeking maybe higher than we estimated, the novel use of data on reported rates of treatment to parameterise the model has led to a more credible model and is justified by the fit to data.

The model is complex and there are many interactions between the parameters. Therefore the values presented here should be considered as a best fitting set of parameters, rather than taken individually. There are limitations to the model structure, e.g. there may be more individual variability between individuals during their sexual life histories than we were able to simulate. There is a trade-off between model complexity and the ability to validate the model with data. More data are needed on sexual life histories as well as further analysis of the sensitivity and robustness of the model assumptions. The advantages of this individual based model over other possible choices are that the history of individuals can be tracked over time, e.g. exposure to infection, previous partners or number of screens. Infection and reinfection events occur within explicitly defined partnerships, which enables partner notification. Finally the model structure is very flexible and additional screening or partner notification strategies and other behavioural patterns or infections can be added.

## Conclusion

The model is applicable to other developed world settings. It is being used to investigate the effectiveness of interventions such as chlamydia screening in England (Turner et al, *submitted*). Modelling is underway to improve understanding of the natural history of pelvic inflammatory disease and estimate the cost-effectiveness of interventions designed to prevent it. The model fitting was as systematic as possible given the limitations of computing time and data. A strength is the use of novel data on past treatment to improve parameter estimates. We therefore believe this model to be a significant improvement in providing a realistic model for use in public health decision-making.

## Abbreviations

Natsal 2000 – National Survey of Sexual Attitudes and Lifestyles 2000

BASHH – British Association of Sexual Health and HIV

GP – General practice

STI – Sexually Transmitted Infection

## Competing interests

The author(s) declare that they have no competing interests.

## Appendix

The proportion of males in each sexual activity group (defined by the number of partnerships in the last year) by age group is assumed to follow a multinomial distribution. The log-likelihood (*L*_*beh*_) of the model given the data and the saturated log-likelihood (*L*_beh_*) are given by:



where *Q*_*ap *_is the number of males (female results not used for final fitting), age group *a *(16–19, 20–24, 25–29, 30–34, 35–39, 40–44) and sexual activity group *p *with a given number of partners (1, 2–3, 4–7, 8+) observed from Natsal, and *y*_*ap *_*and z*_*ap *_are the proportion of males, age group *a *with *p *number of partners, from the Natsal 2000 data and observed in the model, respectively. The deviance is given by:

*Dev*_*beh *_= (- 2*(*L*_*ap *_- *L*_ap_*))

which was minimised to find the best fitting set of behavioural parameters.

The biological parameters were also fitted using maximum likelihood. As the data are binomial the model log likelihood (L_*bio*_) and saturated log likelihood (L_bio _*) are given by:

L_*bio *_= L_*bio_prev *_+ L_*bio_prop*_

L_bio_* = L_bio_prev_* + L_bio_prop_*

The formula is illustrated for L_*bio_prev*_, and is the same for L_*bio_prop*_:



where *I*_*ga *_is the observed number of infected, *S*_*ga *_the observed number of susceptibles, and *x*_*ga *_is the model estimate of the proportion of infected, by gender *g *and age group *a*. For prevalence, *g *(females), by four age groups *a *(16–19, 20–24, 25–29, 30–44) and for the proportion ever treated, *g *(males, females) by two age groups (16–19, 20–24) and the values summed.

The deviance was calculated and minimised in the fitting routine:

*Dev*_*beh *_= (- 2*(*L*_*ap *_- *L*_ap_*)).
